# *In vitro* anti-inflammatory effects of vitamin D supplementation may be blurred in hemodialysis patients

**DOI:** 10.6061/clinics/2021/e1821

**Published:** 2021-02-16

**Authors:** Paulo C. Gregório, Sergio Bucharles, Regiane S. da Cunha, Tárcio Braga, Ana Clara Almeida, Railson Henneberg, Andréa E.M. Stinghen, Fellype C. Barreto

**Affiliations:** I Laboratorio de Nefrologia Experimental, Departamento de Patologia Basica, Universidade Federal do Parana, Curitiba, PR, BR; II Departamento de Medicina Interna, Divisao de Nefrologia, Universidade Federal do Parana, Curitiba, PR, BR; III Programa de Pos-Graduacao em Ciencias da Saude, Pontificia Universidade Catolica do Parana, Curitiba, PR, BR; IV Departamento de Analises Clinicas, Universidade Federal do Parana, Curitiba, PR, BR

**Keywords:** Hemodialysis, Inflammation, Interleukin-1β, hs-CRP, Mineral metabolism, Secondary hyperparathyroidism, Vitamin D

## Abstract

**OBJECTIVES:**

This study aimed to evaluate the potential anti-inflammatory effects of vitamin D supplementation under uremic conditions, both *in vivo* and *in vitro*, and its effects on the parameters of mineral metabolism.

**METHODS:**

Thirty-two hemodialysis patients were randomly assigned to receive placebo (N=14) or cholecalciferol (N=18) for six months. Serum levels of calcium, phosphate, total alkaline phosphatase, intact parathyroid hormone (iPTH), and vitamin D were measured at baseline and after three and six months. The levels of fibroblast growth factor-23 (FGF-23), interleukin-1β (IL-1β), and high-sensitivity C-reactive protein (hs-CRP) were also measured at baseline and at six months. Human monocytes were used for *in vitro* experiments and treated with cholecalciferol (150 nM) and uremic serum. Cell viability, reactive oxygen species (ROS) production, and cathelicidin (CAMP) expression were evaluated using the 3-(4,5-dimethylthiazol-2-yl)-2,5-diphenyltetrazolium bromide assay, dichloro-dihydro-fluorescein diacetate assay, and real time-quantitative polymerase chain reaction, respectively.

**RESULTS:**

Both patient groups were clinically and biochemically similar at baseline. After six months, the levels of vitamin D and iPTH were higher and lower, respectively, in the cholecalciferol group than in the placebo group (*p*<0.05). There was no significant difference between the parameters of mineral metabolism, such as IL-1β and hs-CRP levels, in both groups. Treatment with uremic serum lowered the monocyte viability (*p*<0.0001) and increased ROS production (*p*<0.01) and CAMP expression (*p*<0.05); these effects were counterbalanced by cholecalciferol treatment (*p*<0.05).

**CONCLUSIONS:**

Thus, cholecalciferol supplementation is an efficient strategy to ameliorate hypovitaminosis D in hemodialysis patients, but its beneficial effects on the control of secondary hyperparathyroidism are relatively unclear. Even though cholecalciferol exhibited anti-inflammatory effects *in vitro*, its short-term supplementation was not effective in improving the inflammatory profile of patients on hemodialysis, as indicated by the IL-1β and hs-CRP levels.

## INTRODUCTION

Cardiovascular disease (CVD) and infections are the main causes of mortality in hemodialysis patients (1-3). The synergism between traditional and non-traditional risk factors has been implicated as the main hurdle in preventing mortality and other adverse outcomes among patients (4). Among the non-traditional risk factors, hypovitaminosis D has received an increasing amount of attention (5,6).

Hypovitaminosis D has been consistently associated with CVD, higher risk of infection, and death in patients with chronic kidney disease (CKD) (7,8). Hypovitaminosis D has also been correlated with vascular dysfunction in pre-dialysis CKD patients (9). One possible explanation for these findings is the pleiotropic effects of vitamin D. Previous studies have reported an association between the use of vitamin D receptor activators (VDRAs), namely, calcitriol and paricalcitol, and an improved survival in dialysis patients (4,10). The survival advantage associated with these activators may be partially explained by their role in the regulation of mineral metabolism (11), in the improvement of cardiovascular function (12), and in the immune response of CKD patients (6,13). In addition, VDRAs increase vitamin D receptor (VDR) expression in endothelial and vascular smooth muscle cells (14), in turn facilitating the effects of vitamin D on the cardiovascular and immune system (6,15-17). Despite of these facts, the potential benefits of vitamin D in the cardiovascular system have been challenged. In a large, randomized, placebo-controlled trial, supplementation with vitamin D at a daily dose of 2000 IU, together with omega-3 fatty acid, was not capable of lowering the risk of cardiovascular events among men who were 50 years of age or older and women who were 55 years of age or older (18).

Non-classic actions of vitamin D are being increasingly recognized because of the ubiquitous expression of the VDR in various organs and systems, including hematopoietic cells, such as neutrophils, monocytes, dendritic cells, and lymphocytes (3,6). Vitamin D modulates the immune response through the inactivation of the NF-κB pathway, reducing inflammation and immune cell activation (5,19). Some studies have reported that vitamin D supplementation may increase the 25(OH)-vitamin D levels and reduce the serum levels of inflammatory cytokines, such as interleukin 6 (IL-6), tumor necrosis factor (TNF-α), and MCP-1, in both pre-dialysis and hemodialysis patients (20,21). Furthermore, vitamin D may activate the transcription/production of antibacterial products by epithelial and immune cells, such as cathelicidin (CAMP), which is an antimicrobial peptide that exerts a wide range of effects against numerous pathogenic microorganisms, including gram-positive and gram-negative bacteria. Thus, lower levels of CAMP may facilitate the occurrence of infection-related complications. Interestingly, hemodialysis patients with septicemia and reduced levels of vitamin D and CAMP are at a higher risk of mortality (22).

Importantly, despite the impaired function of the kidneys in hemodialysis patients, the conversion of 25(OH)-vitamin D to active 1,25(OH)-vitamin D can still occur in monocytes due to the presence of the 1α-hydroxylase enzyme in these cells. Thus, both classical and non-classical effects, including anti-inflammatory effects, of 25(OH)-vitamin D supplementation is expected in hemodialysis patients. This study aimed to investigate the potential anti-inflammatory effects of vitamin D supplementation *in vitro*, using a monocyte cell model, and in the clinical setting, in hemodialysis patients with hypovitaminosis D, along with its effects on the parameters of mineral metabolism.

## PATIENT RECRUITMENT AND METHODS

### Patients

Thirty-two patients with stable end-stage kidney disease on hemodialysis were selected from a single dialysis unit (Evangélico Hemodialysis Facility, Curitiba, Brazil). The inclusion criteria were as follows: patients who were on hemodialysis for at least 3 months; patients who were older than 18 years; and patients who exhibited hypovitaminosis D (25(OH)-vitamin D levels<30 ng/mL). The exclusion criteria were as follows: patients who exhibited an active inflammatory or infectious disease; patients with cancer, autoimmune disease, or hepatic dysfunction; patients who were unstable with an expected survival of less than one year; patients who exhibited intact parathyroid hormone (iPTH) levels>600 pg/mL, hypercalcemia (serum calcium>10.3 mg/dL), and/or hyperphosphatemia (serum phosphate>5.5 mg/dL); patients who were previous users or are current users of cinacalcet, nutritional or active forms of vitamin D, anti-osteoporotic medications, glucocorticoids, or immunosuppressive drugs; patients who had a previous history of parathyroidectomy; and patients who were pregnant. The study was reviewed and approved by the Ethical Committee of Pontifícia Universidade Católica do Paraná (CEP/PUC-PR n° 1.682.929). All patients provided signed informed consent.

### Study design

The study was a six-month, prospective, single-blind study, wherein 32 patients with hypovitaminosis D were randomly assigned to receive either cholecalciferol (N=18) or placebo (N=14). All patients in the treatment group received a weekly cholecalciferol dose of 50.000 IU during the first three months. At this time point of the study, the serum level of 25(OH)-vitamin D was measured and the dose of cholecalciferol was adjusted as follows: patients who achieved vitamin D sufficiency (≥30 ng/mL) were switched to a dose of 50.000 IU per month, whereas those whose vitamin D levels remained lower than 30 ng/mL were continued on the same initial dose, that is, 50.000 IU per week. Cholecalciferol was administered for an additional three months, totaling six months of supplementation. Cholecalciferol or placebo was administered after the second dialysis session of the week, under medical supervision to ensure compliance. It was withdrawn upon instances of hypercalcemia (>10.30 mg/dL) or hypervitaminosis D (>150 ng/mL).

Demographic and clinical data, such as age, sex, race, and comorbid conditions, such as hypertension, cardiovascular disease, and cerebrovascular disease, were obtained from the patient’s clinical records. Adjustment of medications and hemodialysis prescriptions were made according to the discretion of the investigating physician, in agreement with the international guidelines on CKD (23).

### Preparation of human serum pool

Healthy and uremic serum pools were prepared and collected according to a previous study (24). Briefly, blood samples (20 mL) were collected in the fasting state before the second hemodialysis session of the week, at baseline (T0), three months (T3), and six months (T6). Blood samples were collected in tubes with anticoagulants and centrifuged at 500 *g* for 10 min, divided into aliquots, and stored at -80°C for subsequent analysis and cell culture experiments.

The healthy/control serum pool consisted of serum samples collected from healthy individuals (N=9) in the fasting state.

The uremic serum pool consisted of serum samples collected at baseline from the hemodialysis patients (N=20). Vitamin D levels in the uremic serum pool were 16 ng/mL.

### Parameters of laboratory testing

Serum levels of total calcium, phosphate, total alkaline phosphatase, iPTH, and 25(OH)-vitamin D were measured at T0, T3, and T6. Serum levels of fibroblast growth factor-23 (FGF-23), interleukin-1β (IL-1β), and high-sensitivity C-reactive protein (hs-CRP) were measured at T0 and T6.

Serum calcium and phosphate levels were measured using a colorimetric assay. Serum iPTH levels were assessed by immunochemiluminescence (reference range: 10-65 pg/mL), while serum levels of 25(OH)-vitamin D were evaluated by radioimmunoassay (DiaSorin Liaison, Vercelli, Italy, with an average intra-assay and inter-assay coefficient of variability of 4% and 6%, respectively).

Serum concentrations of FGF-23 (Immutopics, San Clemente, USA) and IL-1β (R&D Systems, Minneapolis, USA) were measured by enzyme-linked immunosorbent assay. Serum levels of hs-CRP (Abbott, Illinois, USA) were measured by immunoturbidimetry. The measuring ranges for FGF-23, IL-1β, and hs-CRP levels were 0-2200 pg/mL, 0.48-500 pg/mL, and 0-16 mg/dL, respectively. All samples were analyzed simultaneously under standardized experimental conditions in duplicates.

### *In vitro* experiments

#### Monocyte culture and treatment conditions

The monocyte cell line U-937 (CRL-1593.2; ATCC, Manassas, VA, USA) was acquired from a commercial tumor cell line. These cells are considered proxies for circulating monocytes, which are difficult to obtain in satisfactory amounts from whole blood samples. Monocytes were cultured in RPMI 1640 (Gibco, Grand Island, USA) supplemented with 10% fetal bovine serum (Gibco, Grand Island, NY, USA), 100 U/mL penicillin, and 0.1 mg/mL streptomycin (Gibco, Grand Island, NY, USA). As the cells grew in suspension, a concentration of 10^5^ monocytes/mL was maintained in the culture medium every 2 days. The cells were maintained in culture flasks and incubated at 37°C and 5% CO2 conditions. Afterwards, the medium was changed and the cells were incubated with the control (RPMI 1640 supplemented with 10% serum from healthy volunteers, 100 U/mL of penicillin and 50 µg/mL of streptomycin [Gibco, Grand Island, USA]) or uremic medium (RPMI 1640 supplemented with 10% uremic serum from the hemodialysis pool, 100 U/mL of penicillin, and 50 µg/mL of streptomycin) for 6h. Vitamin D and ketoconazole, a 1α-hydroxylase inhibitor, were used at concentrations of 150 nM and 5 µM, respectively (25,26). Four different experiments were performed, wherein each sample was analyzed in duplicate. The mean of each duplicate was used for statistical analysis.

### Cell viability assay

Cell viability was assessed by the 3-[4,5-dimethylthiazol-2-yl]-2,5-diphenyltetrazolium bromide (MTT; Sigma, St. Louis, USA) assay, as previously described (24). Briefly, monocytes were plated in 96-well culture plates at a density of 10^5^ cells per well. After 24h of incubation, the medium was removed and the cells were treated with the following serum pools: control serum (CS); uremic serum (US); uremic serum and vitamin D (Sigma-Aldrich, Missouri, USA; US+Vit. D); uremic serum, vitamin D, and ketoconazole (US+Vit. D+K) for 6h. This medium was then replaced with fresh medium (100 µL/well), and 10 µL of MTT (Sigma-Aldrich, Missouri, USA) solution (5 mg/mL in D-PBS) was added to each well and incubated for 4h at 37°C. Subsequently, the media was removed and replaced with dimethyl sulfoxide (DMSO; Sigma-Aldrich, St. Louis, MO, USA) to dissolve the crystals of the reduced formazan. The absorbance of the samples was measured at 570 nm (Tecan, Männedorf, Switzerland), and all analyses were performed in triplicate.

### Measurement of reactive oxygen species (ROS) production

The ROS-sensitive fluorescent dye 2’,7’-dichlorofluorescein diacetate (DCFH-DA; Sigma, St. Louis, USA) was used to measure ROS production (mostly peroxide). Monocytes (10^5^ cells/well) were seeded in a transparent 96-well plate. After 24h of culture, they were washed with Dulbecco's phosphate-buffered saline (D-PBS) at 37°C and labeled with 1 µM DCFH-DA for 30 min at 37°C. Then, the cells were washed twice with D-PBS, and the different serum pools (CS, US, US+Vit. D, and US+Vit. D+K) were added to each well. Some wells were treated with the antioxidant N-acetyl-cysteine (NAC; 0.2 mM) for 4h prior to treatment with the uremic pools. Fluorescence was immediately measured using a spectrofluorometer (λEx, 492 nm; λEm, 535 nm). Results were expressed as the percentage increase in fluorescence intensity compared to that of the CS-treated cells (27). Six independent experiments were performed, wherein each sample was analyzed in duplicate.

### Measurement of CAMP gene expression by real time quantitative polymerase chain reaction

Total RNA was isolated from the lysed monocytes using TRIzol Invitrogen (Carlsbad, USA). RNA purity and concentration were estimated by measuring the A260 nm/A280 nm absorbance ratio using a NanoDrop 2000 spectrophotometer (Thermo Scientific, Waltham, WA, USA). RNA integrity was analyzed by agarose gel electrophoresis (1%). The mRNA was transcribed into complementary DNA (cDNA) using the High Capacity RNA-to-cDNA Kit (Applied Biosystems, Foster City, CA, USA). cDNA was amplified with specific primers and EvaGreen Master Mix S (Applied Biological Materials, Richmond, BC, Canada) using the Rotor-Gene 6000 thermal cycler (Corbett Research Inc., Mortlake NSW, Australia). Hypoxanthine phosphoribosyl transferase (*HPRT*) was used as a housekeeping gene as described previously (28). The primers used were as follows: human CAMP (forward 5′-TCA CCA GAG GAT TGT GAC TTC AA-3′ and reverse 5′-CCA GCA GGG CAA ATC TCT TG-3′), and human HPRT (forward 5′-GAA CGT CTT GCT CGA GAT GTG A-3′ and reverse 5′-TCC AGC AGG TCA GCA AAG AAT-3′). The relative gene expression levels were calculated using the 2^-ΔΔCT^ method (29). Six independent experiments were performed, wherein each sample was analyzed in duplicate.

### Data analysis

Statistical analyses were performed using the statistical packages JMP (version 8.0; SAS Institute Inc., Cary, NC, USA) and Sigma Stat (version 3.5; Systat software Inc., Erkrath, Germany). Comparisons between groups were performed either using Student’s *t*-test or analysis of variance (ANOVA) for normally distributed variables, and Mann Whitney and ANOVA on ranks for non-parametric variables. The Shapiro-Wilk normality test was used to test normality of the data. Differences with *p* values<0.05 were considered statistically significant.

## RESULTS

### Clinical results

The baseline clinical and laboratory characteristics of the cholecalciferol and placebo group were found to be similar (Table 1). There was no difference in the use of anti-platelet agents (8 patients in the cholecalciferol group and 11 in the placebo group), statins (6 in the cholecalciferol group and 7 in the placebo group), and therapeutic regimens against diabetes (6 patients in each group) between the cholecalciferol and placebo groups.

In the treatment group, the serum levels of 25(OH)-vitamin D were significantly higher at T3 and T6 than at T0, whereas the serum levels of iPTH remained stable throughout the study. Interestingly, all patients achieved normal levels of vitamin D after the first three months of supplementation. In the placebo group, neither 25(OH)-vitamin D nor iPTH serum levels significantly changed throughout the study (Table 2). However, iPTH levels were significantly higher in the placebo group than in the cholecalciferol group at the end of the study (T6) (*p*<0.05). No remarkable change was observed in the serum levels of the other parameters of mineral metabolism, namely, the calcium, phosphate, total alkaline phosphatase, and FGF-23 levels. The serum levels of IL-1β and hs-CRP did not significantly change during the study in either group.

The sole episode of hypercalcemia (Ca>10.3 mg/dL) occurred in the cholecalciferol group at six months. There were nine episodes of hyperphosphatemia (P>5.5 mg/dL), six of which were in the cholecalciferol group. Hyperphosphatemia was easily handled by dietary counseling and adjustment of the dose of the phosphate binder.

There were no significant differences in hemoglobin, hematocrit, and albumin serum levels, white blood cell count, body mass index, and protein catabolic rate between the placebo and cholecalciferol groups during the study (Table 3). 

### Causes of drop out

Eight patients (four patients from each group) did not complete the study. The causes of drop out were death, five patients (three from placebo and two from the cholecalciferol group); renal transplantation, two patients (one from each group); and a change in the dialysis center, one patient (from the cholecalciferol group).

### *In vitro* results

#### Cell viability assay

**Figure 1:**
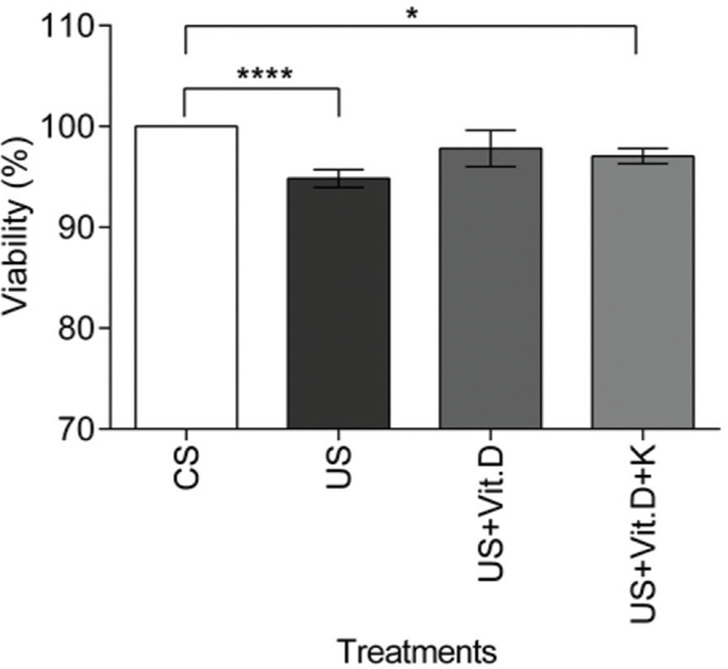
The effect of control serum (CS); uremic serum (US); uremic serum and vitamin D (US+Vit. D); and uremic serum, vitamin D, and ketoconazole (US+Vit. D+K) on monocyte viability. Monocytes (10^5^) were cultured with CS, US, US+Vit. D, and US+Vit. D+K for 24 h and then treated with MTT for 4 h. Cell viability was determined by measuring the absorbance at 570 nm. The viability of the control cells (cells treated with CS and media) was considered 100%. Data are expressed as the mean±SEM of three independent experiments. *p<0.05, US *vs*. US+Vit. D+K and ****p<0.0001, CS *vs*. US.

Cell viability was assessed by the MTT assay. There was a significant decrease in the viability of US- or US+Vit. D+K-treated cells compared to that of the CS-treated cells (*p*<0.05).The US+Vit D treatment induced no change in cell viability, compared to the CS treatment (Figure 1).

### ROS production

**Figure 2:**
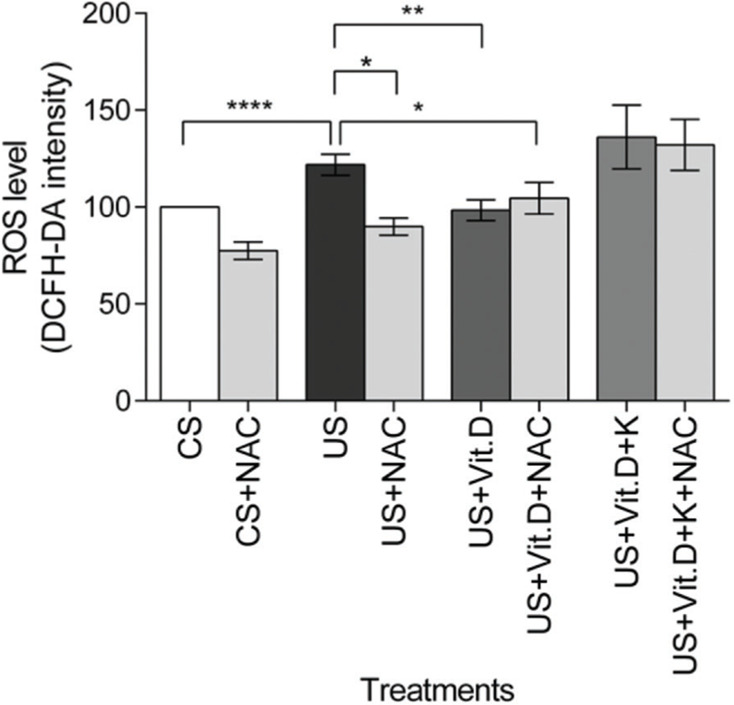
Effect of control serum (CS); uremic serum (US); uremic serum and vitamin D (US+Vit. D); and uremic serum, vitamin D, and ketoconazole (US+Vit. D+K) on reactive oxygen species (ROS) production in monocytes. Monocytes (10^5^) were incubated with 1 µM DCFH-DA in D-PBS at 37°C for 30 min and then treated with CS, US, US+Vit. D, or US+Vit. D+K with or without the antioxidant N-acetyl-L-cysteine (NAC; 0.2 mM). ROS production in CS-treated cells was considered 100%. Data are expressed as mean±SEM of ten independent experiments. *p<0.05, US *vs*. US+NAC and US *vs*. US+Vit. D+NAC; **p<0.01, US *vs*. US+Vit. D; and ****p<0.0001, US *vs*. CS.

The US significantly increased the ROS levels in the cells, compared to the CS treatment (*p*<0.0001). US-induced ROS production was significantly lowered by NAC (US+NAC) and vitamin D (US+Vit. D; *p*<0.01 *vs*. US), as well as by the concomitant use of both NAC and vitamin D (US+Vit. D+NAC) (*p*<0.05, *vs.* US). Finally, the addition of ketoconazole prevented the oxidative effects of vitamin D (Figure 2).

### CAMP gene expression

**Figure 3:**
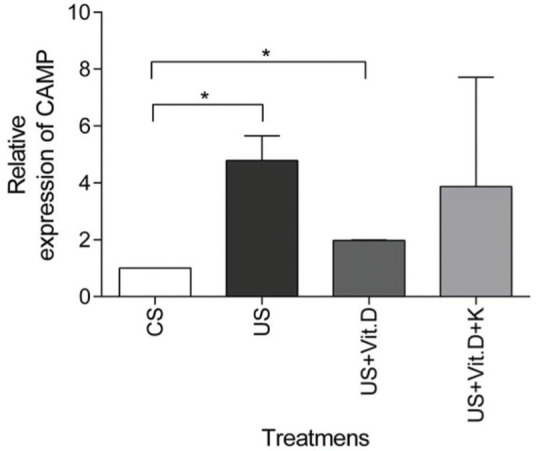
mRNA gene expression of CAMP in monocytes. Monocytes were treated with control serum (CS); uremic serum (US); uremic serum and vitamin D (US+Vit. D); uremic serum, vitamin D and ketoconazole (US+Vit. D+K) for 6 h. Data are expressed as the mean±SEM of five independent experiments. *p<0.05, US *vs*. CS and US+Vit. D *vs*. CS.

US treatment resulted in a significantly higher expression of the CAMP gene (*p<*0.05) in comparison to that induced by CS, whereas CAMP expression was significantly decreased by US+Vit. D treatment (*p*<0.05), compared to the US treatment (Figure 3). Ketoconazole tended to prevent the beneficial effects of vitamin D in uremic conditions, but without resulting in a statistically significant change in the CAMP gene expression (Figure 3).

## DISCUSSION

CKD has been described as an inflammatory condition associated with a higher risk of CVD and infection, both of which are the main causes of mortality among CKD patients (7,30). Interestingly, observational studies have reported that the mortality of CKD patients might be attenuated by VDRAs, suggesting that the vitamin D axis may be a potential modifiable factor of CKD-related complications (31). The effects of vitamin D supplementation on mineral metabolism and its potential pleiotropic actions in the cardiovascular and immune system have been studied in patients with different stages of CKD (4,6,17). We previously demonstrated an association between hypovitaminosis D and inflammation (32), and that vitamin D supplementation might attenuate systemic inflammation and left ventricular hypertrophy in hemodialysis patients (17). In this study, we evaluated the clinical and *in vitro* effects of vitamin D supplementation on inflammation, oxidative stress, and parameters of mineral metabolism in hemodialysis patients and a monocyte cell line (U-937), respectively.

Our study demonstrated that vitamin D supplementation ameliorated hypovitaminosis D, as observed from the third month of follow-up, and serum levels of iPTH did not significantly change in stable hemodialysis patients not receiving other therapies targeting the parathyroids, *i.e.*, VDRAs or calcimimetics. Even though the levels of vitamin D increased in the placebo group, in contrast to the patients who used cholecalciferol, they did not reach the normal range and the serum levels of iPTH tended to increase. The serum levels of calcium, phosphorus, total alkaline phosphatase, and FGF-23 did not significantly change in either group. Despite the fact that the beneficial effect of vitamin D supplementation on the control of secondary hyperparathyroidism has not been uniformly observed in hemodialysis patients (33-35), a recent meta-analysis reported that cholecalciferol may improve the parameters of mineral metabolism in CKD patients (36).

The anti-inflammatory role of cholecalciferol in hemodialysis patients, based on the IL-1β and hs-CRP serum levels, was also evaluated. However, there were no significant changes in the levels of these inflammatory biomarkers. Other studies have not demonstrated beneficial effects of vitamin D supplementation for over 12 weeks on the expression of inflammatory biomarkers, such as TNF-α, IL-6, neutrophil gelatinase-associated lipocalin (NGAL), interferon gamma-induced protein-10 (IP-10), or CAMP, in hemodialysis patients (20) or in overweight individuals (37). Similarly, cholecalciferol supplementation for three months in African Americans did not reduce the levels of C-reactive protein (CRP), IL-6, and IL-10 (38). Other studies have supported the anti-inflammatory effects of vitamin D. A four-week administration of cholecalciferol significantly lowered the hs-CRP levels in older females with vitamin D insufficiency (39). A study has also demonstrated that a one-year supplementation of cholecalciferol in overweight individuals decreased the serum levels of IL-6, but the levels of hs-CRP and TNF-α significantly increased and remained unchanged, respectively (40). Studies in hemodialysis patients have demonstrated that cholecalciferol supplementation may attenuate inflammation in this population (17,41,42). Differences in the characteristics of the study population, such as age or ethnicity, cholecalciferol supplementation dose, length of follow-up, and concomitant use of other medications, may influence the inflammatory status and result in conflicting observations.

In order to investigate the *in vitro* anti-inflammatory effects of vitamin D in an uremic environment, monocyte viability was evaluated under different conditions. We found that uremic serum reduced monocyte viability, whereas vitamin D was able to increase it, and ketoconazole, an inhibitor of 1α-hydroxylase, reversed this beneficial effect of vitamin D. Furthermore, uremic serum increased the production of ROS in monocytes, and this was partially reversed by NAC and vitamin D. Taken together, these results demonstrate the potential *in vitro* benefits of cholecalciferol on monocyte function, such as improvement of cell viability and reduction of oxidative stress. Biomarkers of oxidative stress and inflammation may be found at higher levels in hemodialysis patients, along with increased risk for CVD, death, and other uremia-related comorbidities (1,43,44), and this may result from uremia and dialysis itself. Vitamin D can modulate important pathways of the immune system, such as the toll-like receptor signaling pathway, suppressor of cytokine signaling 1 pathway, and NF-κB pathway in human macrophages (45-47). Recently, Brito et al. described a favorable effect of vitamin D supplementation on the levels of TLR-4, MCP-1, and CAMP in monocytes treated with uremic serum (42).

Interestingly, we observed an increased expression of the CAMP gene after exposure to uremic serum *in vitro*, and this was partially diminished by vitamin D. Grabulosa et al. demonstrated that the serum level of CAMP was higher in hemodialysis patients than that in pre-dialysis CKD patients (47), while other studies have shown that uremia may lower the serum levels of CAMP (20). The effects of vitamin D on CAMP levels seem to be controversial. A 12-week supplementation with cholecalciferol had no effect on the CAMP levels in patients with early CKD (20), whereas active vitamin D was shown to increase the serum levels of this antimicrobial peptide *in vitro* (48,49). A possible explanation for the lack of homogeneity among the observed findings is that previous studies evaluated only the supernatant and/or serum level of CAMP, whereas in our study, we evaluated the transcriptional effects of uremia and vitamin D. Therefore, we hypothesized that post-transcriptional effects may counteract the stimulatory and inhibitory transcriptional effects of uremia and vitamin D, respectively. Most importantly, our findings further indicate that monocytes exposed to the uremic serum pool may still possess functional 1α-hydroxylase. Thus, one may consider that cholecalciferol supplementation may be valuable for CKD patients with hypovitaminosis D. Furthermore, a previous study revealed that dual treatment with cholecalciferol and active vitamin D increased the serum CAMP levels in up to 40% of patients with high parathyroid levels (50). The percentage increase in the serum CAMP and 25(OH)-vitamin D levels was closely correlated. The discrepancy between the *in vitro* and *in vivo* anti-inflammatory effects might also be explained by the complex interactions of external modulating factors - both disease - and therapy-related - in CKD patients subjected to renal replacement therapy. Combining active and native vitamin D analogs, possibly with other approaches to mitigate inflammation, for example, dietary phosphate restriction, anemia, and underlying comorbid conditions, may be the best strategy for the treatment of hypovitaminosis D in hemodialysis patients.

We recognize that our study has some shortcomings, including the relatively low number of patients included, mainly due to the strict inclusion criteria. The duration of follow-up precluded the evaluation of the possible long-term benefits of cholecalciferol on inflammation. Moreover, CAMP expression was not evaluated in the patients and was only evaluated *in vitro*. It would have been interesting to investigate the effect of uremic serum and vitamin D in other cell lines, such as THP-1, and monocytes extracted from the peripheral blood of CKD patients. One possible explanation for the disagreement between the experimental and clinical effects found in our study could be attributed to the differences between tissues and cells. Despite using a pool of uremic serum, *in vitro* samples were collected at one time point, that is, at baseline, while patients were exposed to a myriad of uncontrolled factors that may have altered their serum composition throughout the study, including varying concentrations of uremic toxins such as FGF-23 and phosphate that may interfere with monocyte 1α-hydroxylase activity; this may have precluded the effects of the cholecalciferol supplementation in the clinical setting. Furthermore, as patients with different degrees of hypovitaminosis D, that is, severe deficiency, deficiency, and insufficiency, might have different inflammatory profiles, it would be interesting to investigate the effects of vitamin D supplementation according to the stratum of vitamin D levels in the patients. Unfortunately, our study was not powered for this analysis.

In conclusion, our study demonstrated that cholecalciferol supplementation is an efficient strategy to ameliorate hypovitaminosis D in hemodialysis patients, but with no clear beneficial effects on the control of secondary hyperparathyroidism. Despite exerting beneficial effects against inflammation and oxidative stress, which were demonstrated *in vitro*, cholecalciferol could not ameliorate inflammation in the clinical setting, as indicated by the levels of IL-1β and hs-CRP. Further studies are necessary to establish the role of nutritional vitamin D, its optimal dose, and the length of use, as an anti-inflammatory strategy in dialysis patients.

## AUTHOR CONTRIBUTIONS

Gregório PC, Bucharles S, Cunha RS, Almeida CA, Braga T and Henneberg R carried out the experiments. Gregório PC and Cunha RS performed the statistical analysis. Stinghen AEM and Barreto FC provided advice on the experiments and supported the interpretation of results. Gregório PC, Stinghen AEM and Barreto FC drafted and edited the manuscript. All authors have read and approved the content of the manuscript.

## Figures and Tables

**Table 1:** Clinical and laboratory characteristics of the study population.

	Study Population (N=32)	Placebo (N=14)	Cholecalciferol (N=18)
Age (years)	58.5 (51.25-67.50)	55.5 (50.50-65.25)	59.0 (51.75- 0.25)
Race, Caucasians (%)	93.5	64.2	66.6
Gender, male (%)	51.7	53	50
Arteriovenous access (%)	81	87	75
Etiology CKD (%)			
Diabetes	48	46.7	50
Hypertension	19	13.3	25
Glomerulonephritis	23	26.7	19
Other Phosphate binders (%) Calcium carbonate Sevelamer	103169	13.32971	63367
Time in dialysis (months)	25.5 (10.75-46.50)	27.5 (12.0- 50.25)	24.5 (10.0-45.5)
Ca (mg/dL)	9.0 (8.4-9.9)	8.8 (8.2-9.7)	9.1 (8.4-10.1)
P (mg/dL)	4.3 (3.4-5.4)	4.0 (3.3-4.9)	4.7 (4.0-6.1)
iPTH (pg/mL)	177.5 (63.0-239.8)	194.5 (173.0-266.0)	83.5 (40.1-218.0)
AP (IU/L)	94.5 (71.5-106.5)	96.0 (71.5-150.8)	82.0 (70.9-101.3)
25(OH)D (ng/mL)	19.9 (11.8-23.6)	22.0 (17.2-24.1)	15.2 (10.6-23.1)
FGF-23 (pg/mL)	248.8 (66.6-924.7)	176.2 (22.9-1061.0)	380.3 (112.4-862.0)
IL-1β (ng/mL)	3.2 (0.8-6.6)	1.6 (0.6-4.8)	4.1 (1.1-15.2)
hs-CRP (mg/dL)	0.3 (0.14-1.2)	0.25 (0.14-1.1)	0.44 (0.25-1.2)

Values are expressed as number (percentage) or median (25^th^ to 75^th^ percentile), where appropriate.

Abbreviations: Ca, calcium; P, phosphorus; iPTH, intact parathyroid hormone; AP, alkaline phosphatase; 25(OH)D, 25-hydroxyvitamin D3; FGF-23, fibroblast growth factor-23; IL-1β, interleukin-1b; hs-CRP, high-sensitivity C-reactive protein.

**Table 2:** Evaluation of the laboratory parameters of mineral metabolism during follow-up.

	T0	T1	T2
**Placebo**			
N	14	13	11
Ca (mg/dL)	8.8 (8.2-9.7)	8.5 (8.1-9.1)	9.4 (8.5-9.7)
P (mg/dL)	4.0 (3.3-4.9)	4.7 (3.2-5.5)	3.7 (2.9-6.2)
iPTH (pg/mL)	194.5 (173.0-266.0)	281.0 (229.0-400.0)	232.0 (142.0-452.0)
AP (IU/L)	96.0 (71.5-150.8)	90.0 (72.2-137.0)	82.0 (62.0-141.0)
25(OH)D (ng/mL)	22.0 (17.2-24.13)	19.5 (15.2-24.35)	25.1 (13.7-31.0)
FGF-23 (pg/mL)	176.2 (22.9-1061.0)	-----	390.3 (9.5-1339.0)
IL-1β (ng/mL)	1.6 (0.6-4.8)	-----	1.0 (0.2-2.4)
hs-PCR (mg/dL)	0.25 (0.14-1.1)	-----	0.44 (0.18-1.9)
**Cholecalciferol**			
N	18	14	12
Ca (mg/dL)	9.1 (8.4-10.1)	9.2 (8.9-9.6)	9.1 (8.9-9.5)
P (mg/dL)	4.4 (3.8-5.4)	4.7 (4.0-6.1)	5.1 (3.8-5.9)
iPTH (pg/mL)	83.5 (40.1-218.0)	112.5 (48.7-162.0)^d^	70.0 (53.0-163.0)^e^
AP (IU/L)	82.0 (70.98-101.3)	102.5 (64.0-108.5)	82.0 (58.0-104.0)
25(OH)D (ng/mL)	15.20 (10.65-23.15)	50.1 (39.1-84.3)^a,b,c^	46.1 (39.1-62.75)^a,b,c^
FGF-23 (pg/mL)	380.3 (112.4-862.0)	-----	198.7 (55.91-754.1)
IL-1β (ng/mL)	4.1 (1.1-15.2)	-----	4.8 (0.2-12.35)
hs-PCR (mg/dL)	0.44 (0.25-1.2)	-----	0.57 (0.17-2.0)

Values are expressed as the medians (25^th^ to 75^th^ percentile).

Abbreviations: Ca, calcium; P, phosphorus; iPTH, intact parathyroid hormone; AP, alkaline phosphatase; 25(OH)D, 25-hydroxyvitamin D3; FGF-23, fibroblast growth factor-23; IL-1β, interleukin-1b; hs-CRP, high-sensitivity C-reactive protein. (a) *vs.* T=0 cholecalciferol group, *p*<0.0001; (b) *vs.* T=1 placebo group, ****p<0.001; (c) *vs.* T2 placebo group, *p<0.05; (d) *vs*. iPTH T=1 placebo group, ****p<0.001; (e) *vs.* iPTH T=2 placebo group, ****p<0.001.

**Table 3:** Evaluation of the hematologic and nutritional parameters during follow-up.

	T0	T2
**Placebo**		
N	14	11
Hemoglobin (g/dL)	12.0±1.3	12.1±1.4
Hematocrit (%)	36.5±4.1	35.9±3.9
Albumin (g/dL)	4.1±0.2	4.2±0.3
BMI (kg/m^2^)	28.4±4.6	28.5±5.0
WBC (mm^3^)	7.271±1.576	7.516±1.925
n-PCR (g/kg/day)	1.09±0.20	1.1± 0.10
**Cholecalciferol**		
N	18	12
Hemoglobin (g/dL)	11.8±1.1	11.6±0.7
Hematocrit (%)	35.9±3.4	34.9±2.8
Albumin (g/dL)	3.9±0.2	4.0±0.2
BMI (kg/m^2^)	28.6±7.4	28.9±7.6
WBC (mm^3^)	7.625±2.225	7.730±1.855
n-PCR (g/kg/day)	1.00±0.21	1.02±0.21

Values are expressed as the mean±standard deviation.

Abbreviations: BMI, body mass index; WBC, white blood cell; n-PCR, protein catabolic rate.

T0 = Baseline, T2 = six-month follow-up period.
